# Hydroxyl-Terminated Triazine Derivatives Grafted Graphene Oxide for Epoxy Composites: Enhancement of Interfacial and Mechanical Properties

**DOI:** 10.3390/polym11111866

**Published:** 2019-11-12

**Authors:** Lichun Ma, Yingying Zhu, Guangshun Wu, Xiaoru Li, Chongao Tian, Yuhang Wang, Longyu Xu, Guojun Song

**Affiliations:** 1 Institute of Polymer Materials, Qingdao University, Qingdao 266071, China; zhuyingying9667@163.com (Y.Z.); lixiaoruqdu@126.com (X.L.); tca1196946937@163.com (C.T.); dyhhgf@foxmail.com (Y.W.); 18640475128@163.com (L.X.); 2 Department of Chemical Engineering and Technology, Ludong University, Yantai 264025, China; wugs_hit1@163.com

**Keywords:** graphene oxide, epoxy resin, triazine derivatives, mechanical properties

## Abstract

An effective approach to the fabrication of progressive epoxy nanocomposites by the incorporation of hydroxyl-terminated dendrimers functionalized graphene oxide (GO-TCT-Tris) is reported. The relationship between surface grafting, chemical construction, morphology, dispersion, and interfacial interaction as well as the corresponding mechanical properties of the composites were studied in detail. It was shown that hydroxyl-terminated triazine derivatives have been resoundingly bonded onto the GO surface through covalent bonding, which effectively improved the dispersion and compatibility of GO sheets in epoxy resin. The tensile and flexural tests manifested that the GO-TCT-Tris/epoxy composites exhibited greater tensile/flexural strength and modulus than either the pure epoxy or the GO/epoxy composites. For GO-TCT-Tris (0.10 wt%)/epoxy composite, the tensile strength and elastic modulus increased from 63 ± 4 to 89 ± 6 MPa (41.27%) and from 2.8 ± 0.1 to 3.6 ± 0.2 GPa (28.57%), and the flexural strength and modulus increased from 106 ± 5 to 158 ± 6 MPa (49.06%) and from 3.0 ± 0.1 to 3.5 ± 0.2 GPa (16.67%), respectively, compared to the pure epoxy matrix. Moreover, the fractographic analysis also illustrated the ameliorative interfacial interaction between GO-TCT-Tris and epoxy matrix.

## 1. Introduction

Epoxy resins are typical thermoset plastic with eminent mechanical properties, chemical stability, corrosion resistance, and insulation behavior that can be extensively used in various applications, such as adhesives, coatings, electronic packaging, and the automotive and the aerospace industry [1,2]. However, the intrinsic brittle nature and inferior crack resistance caused by its highly cross-linked structure limit their usefulness as mechanical components [3,4]. Currently, numerous superior nanofillers (nanoparticles, nanotubes, nanofibers, etc.) and their complex as a second phase to mix epoxy resin have been attempted and considered as an effective approach to enhance the strength, rigidity, tenacity, and even vest multi-functional properties [5,6,7].

Graphene and graphene oxide (GO), a novel two-dimensional nanomaterial, has given rise to considerable theoretical and technological researches on polymer composites due to its fantastic and unparalleled physical properties, including a high specific surface area, high fracture strength, elastic modulus, and thermal conductivity [8,9]. It has become an invaluable nanomaterial for polymer reinforcement. However, GO sheets have low compatibility with most polymers and are inclined to aggregations in the polymer matrix owing to the van der Waals force [10,11], which leads to inferior dispersion and exfoliation of GO sheets and inferior interfacial interaction between GO sheets and the matrix, as well as a restriction of stress transfer from the matrix to the GO sheets [12,13]. Therefore, it is important to improve the dispersion of GO sheets in the polymer matrix and interfacial adhesion with the matrix to achieve a satisfactory mechanical performance and applications of graphene/epoxy composites [14].

It has been reported in our recent publications that functionalization of GO to generate derivatives could improve their dispersion and compatibility properties in the resin matrix [15,16,17]. Covalent functionalization of the GO surface is the most valid way to modulate thee chemical/physical features of GO sheets and provide suitable compatibility and dispersion, as well as accelerate the interfacial load transfer from the matrix to the sheet and improve the performances of the GO/polymer composite [18]. For instance, Liu [19] prepared a covalently bonded epoxy monomers (DER332) and curing agents (diamino diphenyl methane (DDM)-grafted GO (DED-GO) hybrid material through a three-step grafting procedure and acquired a higher improvement in tensile strength (30.0% at 0.2 wt% GO-DED) and elongation at break (16.0%) of the epoxy nanocomposite compared with the neat epoxy resin, respectively. Wan et al. [20] covalently grafted GO with diglycidyl ether of bisphenol A (DGEBA), and the DGEBA-f-GO/epoxy composites resulted in a 34% enhancement in tensile strength and 12% in elastic modulus compared with the pure epoxy. Katti et al. [21] functionalized covalently GO with poly (ether ether ketone) (HPEEK). GO-g-HPEEK sheets were equably dispersed in the matrix and exhibited an excellent improvement in the storage modulus (42%), hardness (65%), and fracture toughness (31%) of the epoxy composite at low weight loadings (~0.5 wt %).

These approaches have demonstrated good results, but some deficiencies still existed, such as high cost, high energy consumption, low efficiency, and even GO or graphene structure destruction [22]. In contrast, the covalent functionalization of GO with small molecules is prospective, which has the superiority of high efficiency, inexpensive, easy operation, and non-destructibility for the structure [23]. Ribeiro et al. [24] introduced tetraethylenepentamine (TEPA) onto the GO surface, which resulted in an improvement of 72% in Young’s modulus and 143% in the hardness of the composites containing 0.5 wt.% of GO-TEPA. In a previous work, we constructed a mine dendrimer with low-cost cyanuric chloride (CTC, as the tree trunk) and diethylenetriamine (DETA, as the branch) through a relatively simple and environmentally friendly fabricating process. The dispersion of GO-TCT-DETA sheets in the epoxy matrix remarkably improved and the mechanical performances of the GO-TCT-DETA/epoxy composites were significantly enhanced compared to that of GO/epoxy composites [25]. However, there are few reports focusing on the functionalization of GO with hydroxyl-terminated dendrimers to enhance the dispersion/exfoliation as well as the mechanical performances of epoxy composites.

Low-cost tris(hydroxymethyl)aminomethane (Tris) has distinct polar features with abundant hydroxyl and amine groups, which could react with the active site on the basal planes and edges of GO and could even participate in a curing reaction with epoxy resin [26], which would improve the dispersion of GO in the epoxy matrix and the interfacial properties between GO and the epoxy resin. In this study, CTC and Tris were used to architecture hydroxyl-terminated triazine derivatives via a facile and effective process. The microstructure of Tris-functionalized GO (GO-TCT-Tris) was confirmed by Fourier transform infrared spectroscopy(FTIR), X-ray photoelectron spectroscopy(XPS), X-ray diffraction(XRD), Thermo gravimetric (TG), Transmission electron microscopy(TEM), Scanning electron microscopy(SEM). Epoxy composites with both GO and GO-TCT-Tris sheets were fabricated. The dispersion, interfacial, and mechanical properties (tensile strength, elastic modulus elongation at break, flexural strength, and modulus) as well as the enhancement mechanism of GO-TCT-Tris sheets/epoxy composites were also extensively investigated.

## 2. Materials and Methods

### 2.1. Materials

Graphite powder was offered by Qingdao Tianhe Co. Ltd. (Qingdao, China) Epoxy resin (E-51, epoxy value = 0.48–0.54) was used as the matrix in the composite. Liquid aromatic amine type epoxy resin hardener 3,3-diethyl-4,4-diamino diphenyl methane (H-256) was supplied by Shanghai Macklin Biochemical Co. (Shanghai, China) Sodium nitrate (NaNO_3_), potassium permanganate (KMnO_4_), hydrogen peroxide (H_2_O_2_), cyanuric chloride (TCT), tris(hydroxymethyl)aminomethane (Tris), triethylamine (TEAE), lithium aluminum hydride (LiAlH_4_), and tetrahydrofuran (THF) were acquired from Tanzhi Co. Ltd. (Tianjin, China). Concentrated sulfuric acid (98% H_2_SO_4_) and hydrochloric acid (37% HCl) were bought from Sinopharm Chemical Reagent Co., Ltd. (Qingdao, China) and were analytical grade.

### 2.2. Preparation of GO by Oxidation of Graphite

GO was synthesized by graphite oxidation based on a modified Hummers’ method [27]. About 8 g of nature graphite was added into a 500-mL three-necked flask, then 360 mL of H_2_SO_4_ and 3.75 g of NaNO_3_ were gradually added under stirring in in ice bath. After 30 min, KMnO_4_ was slowly added below 5 °C and reacted for 2 h. Then, the mixture stirred for 17 h at 35 °C, and slowly mixed with 400 mL of H_2_O and maintained for 1 h. After that, the solution was poured into 660 mL of H_2_O and 60 mL of H_2_O_2_, reacted for 20 min and sonicated for 30 min. After standing for 12 h, the suspension was rinsed with 5% HCl and deionized water to obtain a pH value of 7 and was freeze-dried to acquire GO. It should be noted that the addition of KMnO_4_ to sulfuric acid and subsequent addition of graphite to the mixture should be slow enough to keep the temperature below 5 °C during the addition and at least for the next 2 h after the addition. Otherwise, the reaction mixture may explode.

### 2.3. Functionalization of GO by Grafting TCT and Tris

**Figure 1:**
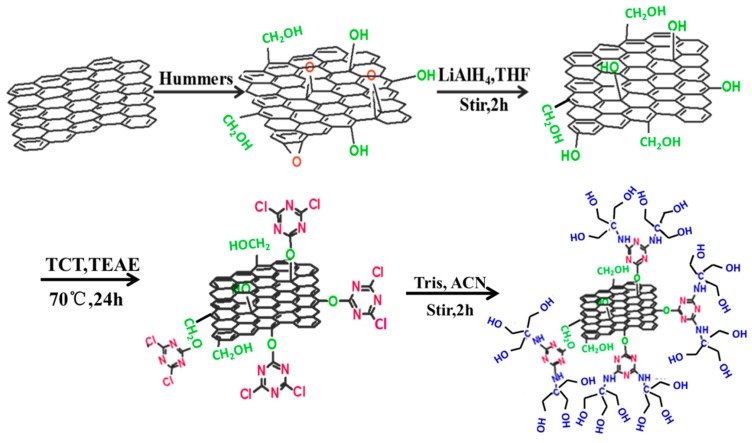
The route of preparation of GO-TCT-Tris.

The prepared GO was firstly dispersed in LiAlH_4_-THF with sonication for 0.5 h and then stirred for 2 h. After that, the mixture was slowly mixed into appropriate water to react the unreacted LiAlH_4_, then washed by ethanol and water, and then dried under vacuum conditions at 80 °C for 6 h to get hydroxylated GO (GO–OH). The GO–OH was dispersed in THF with sonication for 1 h. Under stirring, the mixture was added to TCT and TEAE to absorb the acid produced in the reaction. After stirring at 70 °C for 24 h, the mixture was rinsed thrice by THF to remove the TEAE and ethanol. The suspension was dried under vacuum conditions to get functionalized GO (GO-TCT). The GO-TCT was dissolved in 60 mL of acetonitrile, then Tris and TEAE were added. The mixture was heated and refluxed at 80 °C with stirring for 12 h. Finally, it was rinsed with ethanol and dried to get Tris-functionalized GO-TCT (denoted as GO-TCT-Tris). The preparation route of GO-TCT-Tris is shown in Figure 1.

### 2.4. Fabrication of GO-TCT-Tris/Epoxy Composites

**Figure 2:**
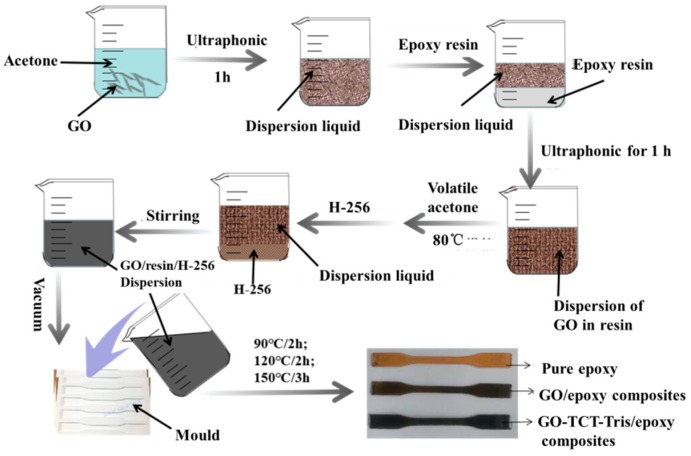
Fabrication schemes of GO-TCT-Tris/epoxy composites.

The fabrication process of GO-TCT-Tris/epoxy composites is presented in Figure 2. Firstly, GO-TCT-Tris sheets were added to acetone and dispersed by sonication for 1 h, and then the epoxy resin was placed into the solution and sonicated for 1 h. Secondly, the mixture was removed into a vacuum oven for 12 h. Then, the curing agent was added under vigorous stirring for 15 min and the bubbles were removed by vacuum distillation. Finally, the mixture was cast into the mold and cured for 2 h at 90 °C, 2 h at 120 °C, and 4 h at 150 °C. For comparison, the neat epoxy, GO/epoxy composites were prepared following the same procedure.

### 2.5. Characterization

Fourier transform infrared spectroscopy (FT-IR, NEXUS, Tokyo, Japan) was conducted to analyze the functional groups on the surface of the graphene and hybrid materials, which were recorded in the wavelength range of 500 to 4000 cm^−1^ and wavenumber at the 4 cm^−1^ resolution. X-ray photoelectron spectroscopy (XPS, ESCALAB 220i-XL, New York, USA) was used to characterize the surface chemical composition and elemental content of the sample. The chemical composition of the sample was investigated by X-ray diffraction (XRD, DX-2700, Tokyo, Japan). The scanning range was from 5° to 45° with a scan rate of 3°·min^−1^. Raman spectra were analyzed using a 633-nm laser and recorded using a LabRAM spectrometer (Berlin, Germany country). TGA-DTG analysis was carried out by STAR SW in the temperature region of 25 to 800 °C and heating rate of 5 °C/min in a nitrogen atmosphere. Scanning electron microscopy (SEM, JSM-7800F, New York, USA) was carried out to investigate the surface morphology of samples. The dispersity of GO in the matrix was observed by transmission electron microscopy (TEM, G2F30, Boston, USA) and optical microscopy. The tensile and flexural tests were examined on a universal testing machine (Al-7000, changchun, China) with a crosshead speed of 2 mm min^−1^ and gauge length of 60 mm. In addition, the specimen dimensions were 80 mm (long) × 10 mm (wide) × 4 mm (thick). Each specimen was tested 5 times to calculate the average value.

## 3. Results

### 3.1. Characterization of GO

**Figure 3:**
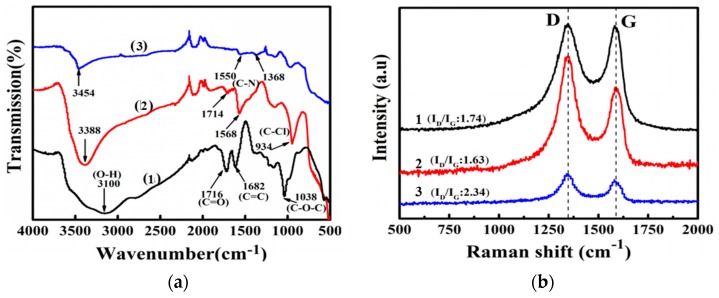
(**a**) FTIR and (**b**) Raman spectra of (1) GO, (2) GO-TCT, and (3) GO-TCT-Tris.

The surface groups on the surface of functionalized GO sheets were measured by FTIR, as shown in Figure 3a. The absorption peak of GO mainly contained stretching vibrations of O–H from the hydroxyl and carboxyl groups at 3100 cm^−1^, C=O from carboxyl at 1716 cm^−1^, C=C from the aromatic ring at 1682 cm^−1^, and C–O–C from epoxy at 1038 cm^-1^ in Figure 3a (1), which demonstrates a large number of oxygen-containing functional groups on the surface of GO and is consistent with the literature [28]. After TCT grafting, the spectrum of GO–TCT showed three new peaks at 1714, 1568, and 934 cm^-1^ corresponding to the C–N bond of the triazine ring and the C–Cl stretching vibrations in Figure 3a (2). After the Tris grafting, the absorption peak of C–Cl disappeared in Figure 3a (3), which was attributed to the reaction of Tris with Cl of cyanuric chloride. These results preliminarily indicated that CTC and Tris were successfully grafted onto the GO surface.

The internal structure of the different sheets was characterized by Raman spectroscopy. The Raman diffraction spectrum of GO and its derivatives mainly includes two bands, D band (1334 cm^−1^) and G band (1585 cm^−1^), as shown in Figure 3b. The relative intensity of the D band reflects the degree of disorder in the crystal structure of the graphene [29]. The relative intensity of the G band represents a first-order scattering E2g vibrational pattern, which characterizes the carbon sp^2^ bond structure [30]. Therefore, the intensity ratio of I_D_/I_G_ is a measured way of disordered graphite. The *I*_D_/I_G_ ratio of GO was 1.74, manifesting that there were some structural defects. The I_D_/I_G_ of GO-TCT dropped down to 1.63 because the TCT had a stable six-membered heterocyclic ring structure that filled up the *sp^2^* area of GO. After Tris modification, the I_D_/I_G_ value of GO-TCT-Tris increased slightly to 2.34, which was because some chemical bonds and hydrogen bonds were formed on the GO surface [31]. The grafting of TCT and Tris onto the GO surface increased its active sites, but the sp^2^ structure of graphene did not show severe damage during the modification process.

**Figure 4:**
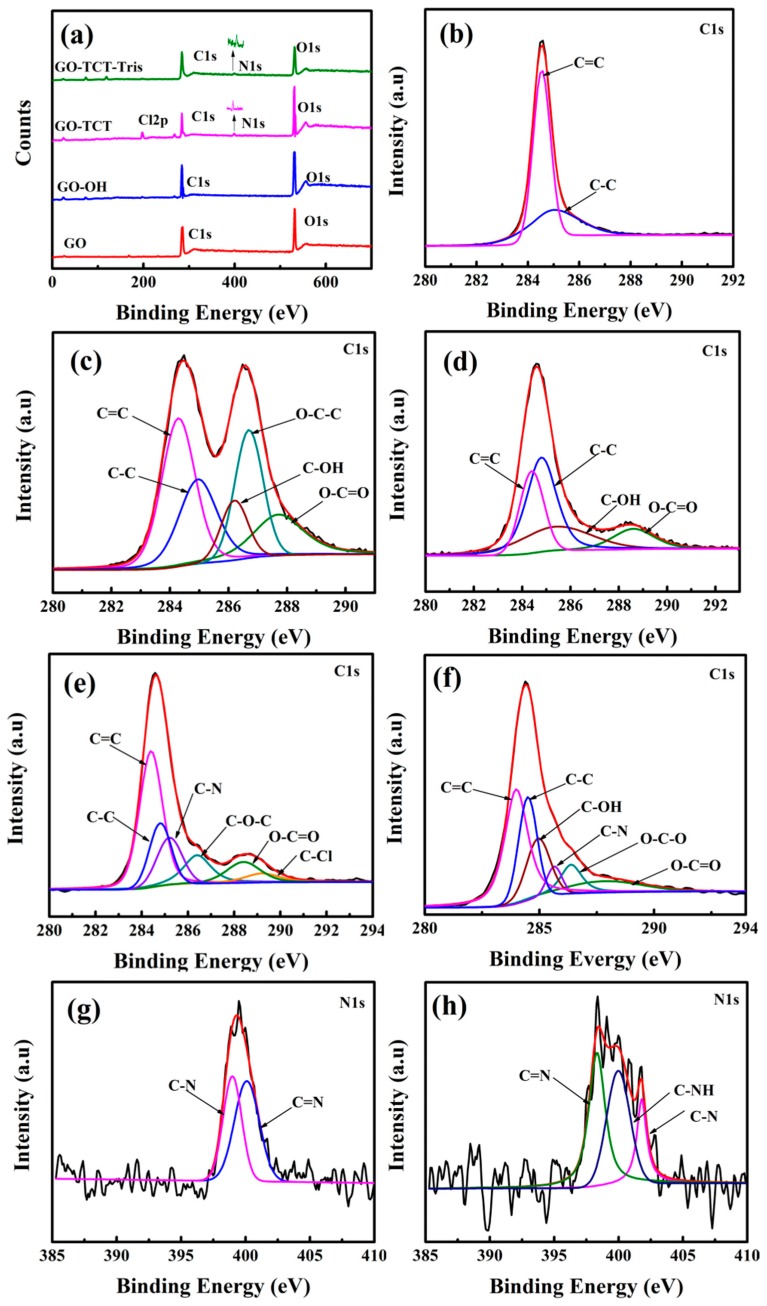
(**a**) XPS spectra and C1 XPS spectra of (**b**) graphite, (**c**) GO, (**d**) GO-OH, (**e**) GO-TCT, and (**f**) GO-TCT-Tris and N1s XPS spectra of (**g**) GO-TCT and (**h**) GO-TCT-Tris.

The surface elements and functional groups of GO and functionalized GO were further demonstrated by XPS, as shown in Table 1 and Figure 4. The peaks of GO were observed as two peaks: O1s (531.93 eV) and C1s (284.61 eV), and the composition of O and C was 40.39% and 59.61%. After TCT functionalization, some new peak Cl2p (197.74 eV) and N1s (399.30 eV) elements appeared; the composition was 4.71% and 2.17%. The N/C and Cl/C atomic ratio increased significantly to 0.045 and 0.098 compared with the previous samples. Respectively, these indicated that TCT adhered to the GO surface. After Tris grafting, the disappearance of the Cl2p peak (197.7 eV) and the small increase of the N1s (399.3 eV) peak from 2.17% to 3.73% was observed. The Cl/C atomic ratio decreased to 0.004 and the N/C atomic ratio increased to 0.066, implying that the reaction proceeded between the chlorine of TCT and the amine groups of Tris.

The high-resolution C1s and N1s spectrum was used to estimate the function groups. The C1s signal peak of GO presented C=C (~284.3 eV), C-C (~284.7 eV), C–OH (~285.6 eV), C–O–C (~286.8 eV), and O–C=O (~288.6 eV) in Figure 4c, which came from the aromatic ring and the oxygen-containing functional groups on the GO surface [31], respectively. For GO–OH (Figure 4d), the content of C–OH continued to increase and those of O–C–O and O–C=O decreased at the same time, indicating that GO was successfully reduced by LiAlH4. For GO-TCT, the peak of C-N (~285.8 eV) and C–Cl (~389.2 eV) was observed, presented in Figure 4e, which was attributed to the reaction between TCT and oxygen-containing functional groups on the GO surface [32]. Compared with GO-TCT, the C–Cl peak disappeared, and the C-OH peak significantly increased in the C1s fitting peak of GO-TCT-Tris (Figure 4f), signifying the covalent bonding of TCT and Tris onto the GO sheets’ surface.

N1s spectra were used to further illustrate the chemical reaction of Tris between the GO-TCT surfaces, as exhibited in Figure 4g,h. For GO-TCT, the N1s fitting peaks of C–N and C=N from the TCT molecule were observed at ~399.0 and ~400.1 eV, which was derived from the triazine ring on TCT. The N1s spectra of GO-TCT-Tris showed a new peak at ~399.5 eV, corresponding to the binding energy of the secondary amine (C-NH) [33]. These results also provided evidence for the successful grafting of TCT and Tris onto the GO surface.

**Figure 5:**
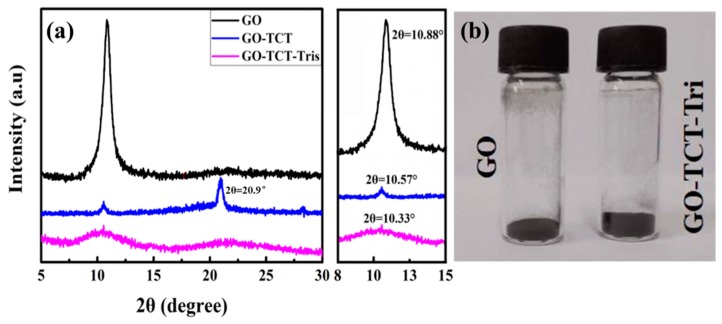
(**a**) XRD scans of GO, GO-TCT, and GO-TCT-Tris, (**b**) volume of GO and GO-TCT-Tris.

The exfoliation level of GO, GO-TCT, and GO-TCT-Tris was characterized by XRD, as shown in Figure 5a. The peak value of GO was 10.86°, corresponding to an interlamellar spacing of 0.81 nm. This was due to the existence of oxygen-rich groups on both sides of the sheets and water molecules trapped between the sheets [34]. After partial reduction and TCT modification, the GO-TCT presented a weak diffraction peak at 10.57°and a strong diffraction peak at 20.9°, corresponding to an interlamellar spacing of 0.42 nm, which was ascribed to the structural defects and the triazine ring of TCT compensating for the GO [35]. Upon covalent functionalization with Tris, the XRD spectrum of GO-TCT-Tris sheets had no obvious diffraction peak and became flat, manifesting that the GO sheets were exfoliated absolutely after the surface functionalization. The covalent grafting of Tris on the GO surface increased the interlayer space between the GO sheets. They stacked together in a loose manner [36,37], as verified by Figure 5b, and the volume of GO-TCT-Tris was greater than that of GO when they had the same quality.

### 3.2. Morphologies of GO Sheets

**Figure 6:**
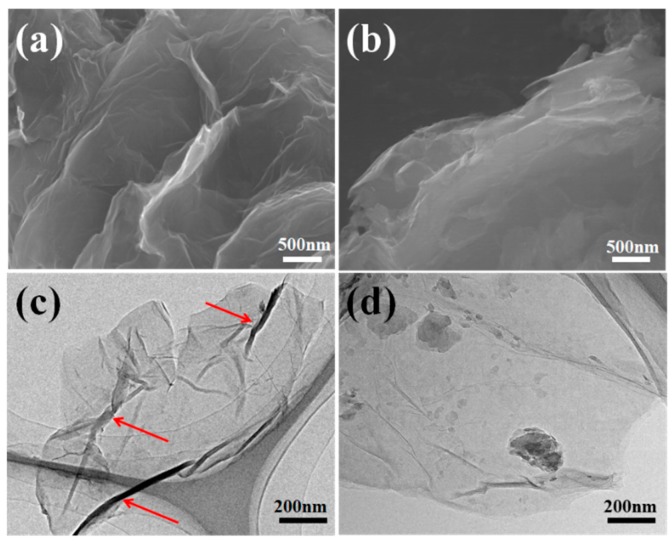
SEM image: (**a**) GO; (**b**) GO-TCT-Tris; Transmission electron microscopy (TEM) image: (**c**) GO; (**d**) GO-TCT-Tris.

The structural morphology and surface characteristics of GO derivate were observed through SEM and TEM, as displayed in Figure 6. The GO sheets (Figure 6a) showed severe aggregates with smooth surfaces, which was caused by the huge surface energy. The edges of the GO-TCT-Tris sheet exhibited many wrinkles with a loose structure, as shown in Figure 6b, which was because the grafted hydroxyl-terminated dendrimers with a network architecture prevented the formation of aggregates. This was in accordance with the slight increase in the I_D_/I_G_ values and layer spacing as mentioned in Raman and XRD.

The TEM image of GO derivate sheets presented a natty and transparent surface with a few thin ripples, as shown in Figure 6c, which was due to the thermodynamic stability of the 2D membranes of GO. However, the GO-TCT-Tris sheets in Figure 6d show well-dispersed and exfoliated states on the mica surface, with some clouds distributed uniformly on the GO sheet surface, suggesting the successful bonding of TCT and Tris on the GO surface, which is often credited as a favorable medium for strong interfacial interaction with the polar epoxy matrix. Moreover, the randomly oriented rough surface of the GO-TCT-Tris sheet could be considered as an effective reinforcing filler [38].

### 3.3. Thermal Stability of GO Sheets

**Figure 7:**
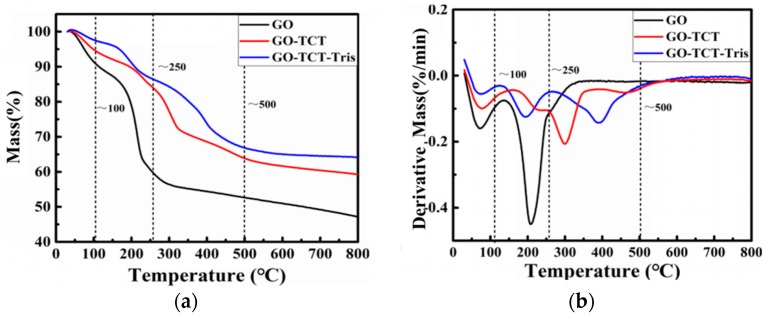
(**a**)TGA and (**b**) DTG curves of the GO, GO-TCT, and GO-TCT-Tris.

To ascertain the thermal stability of GO, GO-TCT, and GO-TCT-Tris as well as the relevant information of the TCT and Tris grafted to the GO sheets [39], TGA and DTG were carried out and are exhibited in Figure 7 and Table 2. The mass loss for the three samples below 100 °C should be attributed to water dehydration. As testified by the FTIR and XPS spectrum, abundant oxygen-containing groups existed on the GO surface, and the major mass loss range of GO was between at 200 and 250 °C. The mass loss of GO-TCT was only about 33% at 800 °C, manifesting the enhanced thermal stability of GO-TCT because some oxygen-containing groups were removed after reduction by LiAlH_4_, manifesting that the TCT molecules grafted on the GO surface, preventing decomposition of the carbon skeleton of GO. Comparatively, for GO-TCT-Tris, at 800 °C, the total mass loss was about 40%; Based on the TGA result, the mass fraction of Tris molecules grafted on the GO sheets could be acquired by calculation, which was ~7%.

### 3.4. Dispersion of GO Derivate Sheets in Solvent and Epoxy Matrix

**Figure 8:**
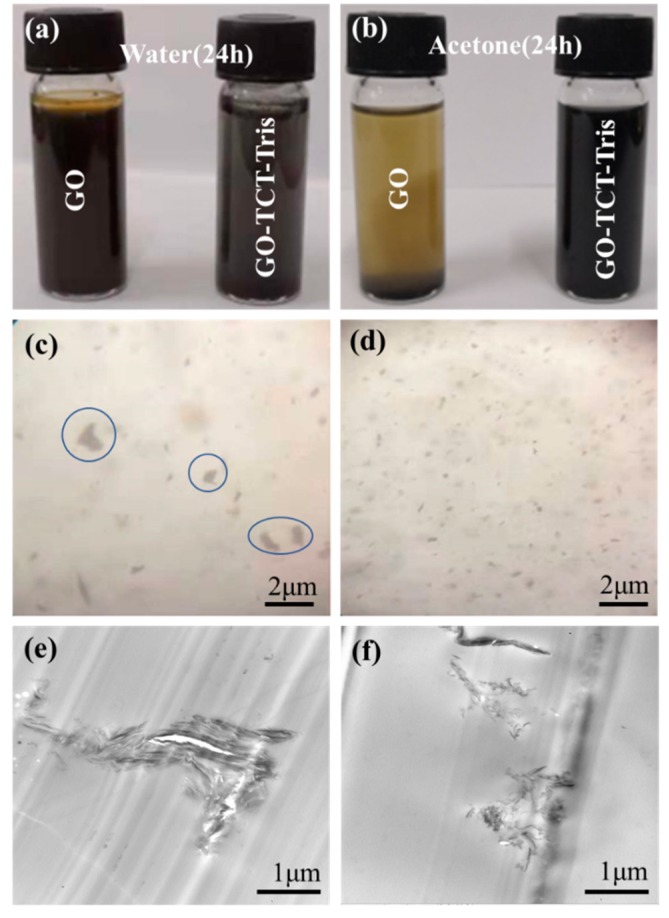
Dispersion of GO and GO-TCT-Tris in water (**a**) and acetone. (**b**) Microscope and TEM images of epoxy composites containing: (**c**,**e**) GO, (**d**,**f**) GO-TCT-Tris.

Figure 8a and Figure 8b show the digital photographs of GO and GO-TCT-Tris after standing for 24 h in a concentration of 1 mg/mL after sonication in different solvents. It can be seen that the GO and GO-TCT-Tris is readily dispersed in water, emerging as the color of brownish yellow and pitch black, respectively. However, the modification of GO by TCT and Tris has a significant influence on its dispersibility in acetone. Untreated GO precipitated in acetone after 24 h, on the other hand, while GO-TCT-Tris remained stable in acetone after 24 h. This indicated that the compatibility of GO-TCT-Tris was improved, which might increase its dispersibility in the polymer matrix [40].

It is well known that nanofillers (such as GO and carbon nanotube) with a high specific surface area are inclined to attract each other to form clusters even within the uncured polymer owing to the van der Waals forces and Coulomb attractions [41]. The dispersion of GO in polymers is a major challenge for nanocomposites in maintaining a stable dispersion and avoiding cluster formation. In addition, the high temperature in the curing process could serve as a driving force to cause GO sheets to reagglomerate [42,43]. To further assess the dispersion of GO and GO-TCT-Tris sheets (with 0.10 wt%) in the epoxy composites, microscope and TEM images of nanocomposites were performed, as presented in Figure 8c. Many agglomerates of the GO sheets were exhibited in the epoxy matrix, as punctuated with blue circles. The TEM image in Figure 8e also ascertained the agglomeration of GO sheets in the epoxy composites. Clearly, although the exfoliation of GO showed a fine degree (Figure 5a), it was inevitable that the GO sheets would tend to agglomerate in the epoxy matrix. Comparatively, for TCT- and Tris molecule-functionalized GO, the dispersion of GO-TCT-Tris became much better (shown in Figure 8d and f). In addition, GO-TCT-Tris sheets seemed to be in a relatively loose state in the epoxy matrix compared to the clusters of GO sheets (shown in Figure 8f and Figure 8e), which was because the grafted hydroxyl-terminated triazine derivatives reduced the mobility of the graphene sheets in the polymer matrix, thereby preventing the dispersed sheets from attracting each other to form re-agglomerates [44]. This result suggested that the Tris functionalization is really effective in improving the exfoliation and dispersion of GO sheets in epoxy matrix, even during the curing process, which would favor stress transfer from the polymeric matrix to the GO sheets and improve the mechanical properties.

### 3.5. Mechanical Properties of Composites

**Figure 9:**
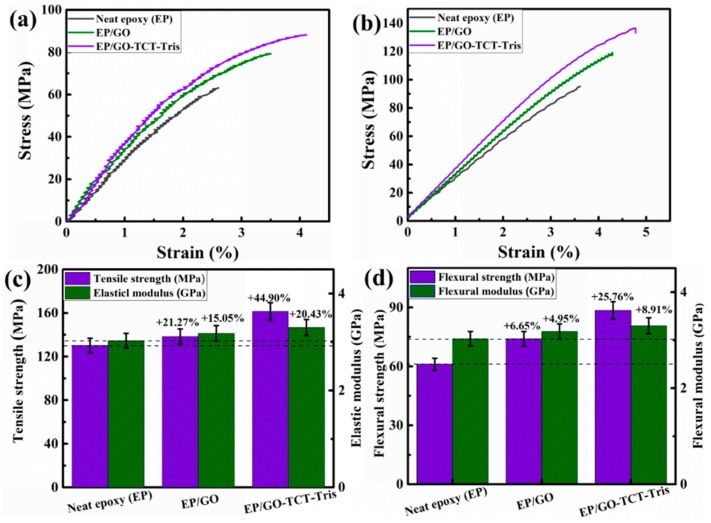
Mechanical performances of epoxy and its nanocomposites: (**a**,**b**) stress–strain curves of the tensile and flexural test, and (**c**,**d**) strength and modulus.

Representative stress–strain curves for neat epoxy and its nanocomposites, along with the results of tensile and flexural testing, including tensile strength, elastic modulus, elongation at break, flexural strength, and modulus, are listed in Figure 9 and Table 3. The tensile strength, elastic modulus, and elongation at break of nanocomposites (0.10 wt% GO loading) increased by 26.98%, 14.29%, and 26.92% compared to pure epoxy. For 0.10 wt% GO-TCT-Tris/epoxy composite, the tensile strength, elastic modulus, and elongation improved by 41.27%, 28.57%, and 61.54% compared with pure epoxy resin, which demonstrated that the GO-TCT-Tris/epoxy composite obtained a better effectiveness than the GO/epoxy composites. The flexural strength and modulus of the modified GO/epoxy composite showed a considerable improvement of 7.48% and 2.94% compared with the GO/epoxy composite. The improved mechanical performances of the GO-TCT-Tris/epoxy composite could be ascribed to the improved exfoliation and dispersion as well as strong interface quality, which were conducive to facilitating the stress transfer from the epoxy matrix to the GO derivative sheets, thus prominently improving the tensile and flexural properties.

**Figure 10:**
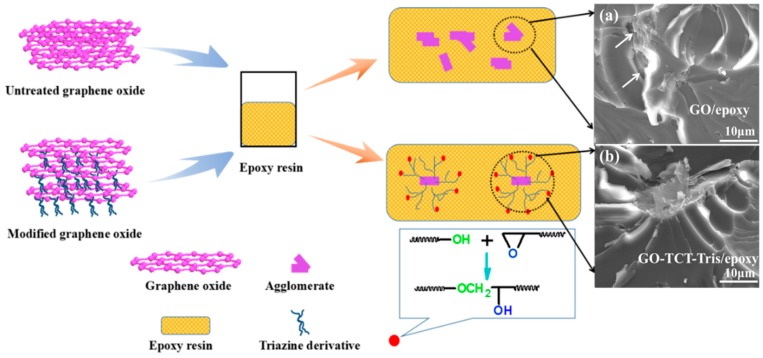
Representation of epoxy nanocomposites and SEM images of cross-sectional fracture surfaces of (**a**) GO/epoxy composite and (**b**) GO-TCT-Tris/epoxy composite after tensile testing.

The interface quality improvement of the GO-TCT-Tris/epoxy composites could be attributed to two aspects: (1) Strong covalent bonding between hydroxyl-terminated triazine derivatives and the GO sheet resulted in the quantity of linked peripheral hydroxyl groups increasing in the presence of hydrogen bonds, which could further react with the epoxy resin, thus forming chemical bonding in the interface to increase the interfacial adhesion and compatibility with the epoxy matrix. Moreover, the hyperbranched triazine derivatives not only enlarged the specific surface area but also had more interspace in its inner and outer, which were penetrated by the epoxy matrix to generate strong mechanical interlocking. Figure 10 shows the reaction diagram between hydroxyl-terminated triazine derivatives and the epoxy matrix, in which strong interfacial bond and mechanical interlocking would be speculated to take place between hydroxyl-terminated triazine derivatives and epoxy molecular chains, effectively facilitating the GO sheets’ exfoliation.

To further estimate the dispersion and interface of GO-TCT-Tris/epoxy composite nanocomposites, the cross-sectional fracture surfaces of the specimens after tensile testing were observed by SEM. For 0.10 wt% GO/epoxy composites, there were many ripples structures on the fracture surface (Figure 10a), which indicated the dispersion was uneven (Figure 10a) and this was in accordance with the microscope and TEM observations. In addition, there were some distinct gaps on the surface, signifying the poor interfacial adhesion between the GO and epoxy matrix. Comparatively, the GO-TCT-Tris/epoxy composites, as shown in Figure 10b, presented numerous smaller ripples with some ribbons and river lines on the cross-sectional fracture surface and some GO-TCT-Tris sheets appeared well dispersed in the epoxy matrix, with no evident aggregation of the sheets. The formation of ripples was accompanied by the generation of new fracture surfaces [45,46], and much more fracture energy would be consumed for GO-TCT-Tris/epoxy composites. This signified that the interface adhesion between GO-TCT-Tris and the epoxy matrix was stronger than that between GO and the epoxy matrix.

## 4. Conclusions

In summary, GO functionalization with low-cost hydroxyl-terminated dendrimer was testified to improve the dispersion/exfoliation and compatibility in epoxy resin. The tensile and flexural strength of GO-TCT-Tris/epoxy composites was significantly enhanced (increased by 41.27% and 49.06%) compared with that of the neat epoxy. The fracture surface morphologies demonstrated that the hydroxyl-terminated dendrimers’ functionalization contributed to better dispersed/exfoliated sheets and stronger interfacial interaction between GO sheets and the epoxy matrix, which facilitated effective stress transfer from the matrix to the GO-TCT-Tris sheets.

## Figures and Tables

**Table 1:** Surface chemical elemental of GO, GO-OH, GO-TCT, and GO-TCT-Tris.

Sample	Composition (%)					
	C	O	N	Cl	N/C	Cl/C
GO	59.61	40.39	—	—	—	—
GO-OH	61.11	38.89	—	—	—	—
GO-TCT	48.22	44.90	2.17	4.71	0.045	0.098
GO-TCT-Tris	56.18	39.89	3.73	0.25	0.066	0.004

**Table 2:** Mass loss values obtained from TGA curves for various samples.

Sample	Mass loss (%) (0–250 °C)	Mass loss (%) (250–500 °C)	Mass loss (%) (500–800 °C)	Residue (%)
GO	40.9	6.3	6.3	46.5
GO-TCT	16.3	20.0	4.3	59.4
GO-TCT-Tris	13.1	19.4	3.3	64.2

**Table 3:** Mechanical performances of epoxy nanocomposites.

Samples	Tensile Strength (MPa)	Elastic Modulus (GPa)	Elongation at Break (%)	Flexural Strength (MPa)	Flexural Modulus (GPa)
Neat epoxy (EP)	63 ± 4	2.8 ± 0.1	2.6 ± 0.3	106 ± 5	3.0 ± 0.1
EP/GO (0.1%)	80 ± 5	3.2 ± 0.1	3.3 ± 0.4	147 ± 3	3.4 ± 0.1
EP/GO-TCT-Tris (0.1%)	89 ± 6	3.6 ± 0.2	4.2 ± 0.5	158 ± 6	3.5 ± 0.2
